# Feasibility of Human Platelet Lysate as an Alternative to Foetal Bovine Serum for In Vitro Expansion of Chondrocytes

**DOI:** 10.3390/ijms22031269

**Published:** 2021-01-28

**Authors:** Ling Ling Liau, Muhammad Najib Fathi bin Hassan, Yee Loong Tang, Min Hwei Ng, Jia Xian Law

**Affiliations:** 1 Physiology Department, Faculty of Medicine, Universiti Kebangsaan Malaysia Medical Centre, Jalan Yaacob Latif, Kuala Lumpur 56000, Malaysia; liaujessy88@gmail.com; 2 Centre for Tissue Engineering and Regenerative Medicine, Faculty of Medicine, Universiti Kebangsaan Malaysia Medical Centre, Jalan Yaacob Latif, Kuala Lumpur 56000, Malaysia; najibfathi93@gmail.com (M.N.F.b.H.); angela@ppukm.ukm.edu.my (M.H.N.); 3 Pathology Department, Faculty of Medicine, Universiti Kebangsaan Malaysia Medical Centre, Jalan Yaacob Latif, Kuala Lumpur 56000, Malaysia; tangyl@ppukm.ukm.edu.my

**Keywords:** chondrocytes, cartilage, osteoarthritis, foetal bovine serum, human platelet lysate

## Abstract

Osteoarthritis (OA) is a degenerative joint disease that affects a lot of people worldwide. Current treatment for OA mainly focuses on halting or slowing down the disease progress and to improve the patient’s quality of life and functionality. Autologous chondrocyte implantation (ACI) is a new treatment modality with the potential to promote regeneration of worn cartilage. Traditionally, foetal bovine serum (FBS) is used to expand the chondrocytes. However, the use of FBS is not ideal for the expansion of cells mean for clinical applications as it possesses the risk of animal pathogen transmission and animal protein transfer to host. Human platelet lysate (HPL) appears to be a suitable alternative to FBS as it is rich in biological factors that enhance cell proliferation. Thus far, HPL has been found to be superior in promoting chondrocyte proliferation compared to FBS. However, both HPL and FBS cannot prevent chondrocyte dedifferentiation. Discrepant results have been reported for the maintenance of chondrocyte redifferentiation potential by HPL. These differences are likely due to the diversity in the HPL preparation methods. In the future, more studies on HPL need to be performed to develop a standardized technique which is capable of producing HPL that can maintain the chondrocyte redifferentiation potential reproducibly. This review discusses the in vitro expansion of chondrocytes with FBS and HPL, focusing on its capability to promote the proliferation and maintain the chondrogenic characteristics of chondrocytes.

## 1. Introduction

Having to go through daily chores and activities can be very stressful when the joints involved in daily locomotion cannot move as smoothly as it should and causing pain. This situation occurs more often with age due to imbalance between cartilage resorption and production as well as accumulation of injury and damage to the cartilage that serves as a cushion between two bones. The prevalence of symptomatic knee osteoarthritis (OA) among people aged 60 years and above is approximately 10–15% in the United States with women more prone to it compared to men [1]. This percentage is expected to further escalate in future due to the aging population, the increasing number of people active in extreme sports and the obesity epidemic.

Several treatment modalities are available with the current knowledge and technology for the management of OA. Treatment for OA can be divided into non-pharmacological, pharmacological and invasive interventions [2]. Non-pharmacological treatments include education, controlled exercise, and lifestyle changes, while the pharmacological treatments include paracetamol, non-steroidal anti-inflammatory drugs (NSAIDs), topical analgesics, and hyaluronic injection. On the other hand, the invasive interventions include osteochondral transplantation, microfracture, microdrilling, total knee replacement, and autologous chondrocyte implantation (ACI) [3]. In practice, treatment of OA starts with the less invasive options before proceeding to more invasive therapies. 

ACI is a relatively new therapy introduced to repair the damaged knee cartilage. ACI is a two-stage operative procedure which involves the biopsy of cartilage tissue for chondrocyte expansion in vitro, followed by transplantation of the expanded cells to the affected joints under the periosteum or a synthetic graft [4,5]. ACI can reduce joint pain and promote cartilage regeneration. The implanted chondrocytes are expected to secrete the cartilage extracellular matrix (ECM) to repair the defect [6]. 

Human platelet lysate (HPL) has been identified as a potential replacement for foetal bovine serum (FBS) for the expansion of chondrocytes. HPL is more suitable for the clinical expansion of chondrocytes in the current good manufacturing practice (cGMP) facility as it is safer with no risk of animal pathogen transmission and carryover of animal protein that may elicit immune response [7]. Importantly, HPL is economical and will not increase the production cost. Similar to FBS, HPL is rich in biological factors that are essential in maintaining chondrocyte survival and proliferation in vitro [8]. 

The current review briefly discusses the anatomy and physiology of cartilage, pathophysiology of OA, and ACI with emphasis given to the in vitro expansion of chondrocytes using FBS and HPL.

## 2. Cartilage Anatomy and Physiology

Cartilage is a specialized elastic connective tissue wrapping the bony articular surface. Cartilage supply a smooth, lubricated surface for articulation and to enable the conveyance of weights with a minimum frictional coefficient [9]. Together with synovial fluid, cartilage support frictionless motion of the joint. Cartilage tissue is hypocellular, aneural, alymphatic, and avascular [10]. Cartilage is composed of chondrocytes which are responsible for the production, organization, and maintenance of the cartilage ECM principally consists of water and macromolecules, including collagens, proteoglycans, and noncollagenous proteins [11]. Chondrocytes are called chondroblasts before they surround themselves with matrix. Chondrocytes arise from mesenchymal stem cells and makes about 2% of the total volume of articular cartilage [9]. As nutrition supply and waste removal of cartilage tissue are dependent on diffusion, which is a slow process, chondrocytes have low metabolic rate and heal slowly after injury [12]. 

Chondrocytes at different zones of the cartilage vary in number, shape and size. In the superficial zone, the chondrocytes appear to be flatter and smaller and generally have a greater density compared to the cells deeper in the matrix. Each chondrocyte provides a unique microenvironment and is accounted for the turnover of the ECM in its immediate vicinity. This microenvironment confines the chondrocyte within its own matrix and prevents migration of the cells to adjacent sites of cartilage. Chondrocytes can sense the changes in ECM structure and react by modulating the matrix anabolism and catabolism and remodelling as the cells substitute the matrix macromolecules lost through degradation [9].

Cartilage is categorized into hyaline cartilage, elastic cartilage and fibrocartilage based on its ECM composition [13]. Generally, hyaline cartilage is the one that is found in joints and helps to ease joint movement by minimizing the friction. 

## 3. Osteoarthritis

OA is a common yet treatable joint disorder. Development of OA is multifactorial as it can be due to age, obesity, gender, knee injury, over and repetitive use of knee, muscle weakness and joint laxity [1]. The risk of OA has been reported to increase in elderly, women and people who are obese, physically inactive and with history of knee trauma [14,15]. In addition, OA is also related to genetic and occupational risk factors [16]. Individual with abnormal joint anatomy, joint instability, inadequate muscle strength or disturbance of joint or muscle innervation also has increased risk of OA [17]. 

OA can manifest with joint pain when used while stiffness is felt at rest. Restricted range of motion due to pain will greatly reduce the patient’s quality of life [18]. In normal adult, cartilage degeneration in OA happened in two phases, i.e., biosynthetic phase and degradative phase [19,20]. In biosynthetic phase, a variety of anabolic cytokines and growth factors such as bone morphogenic proteins (BMPs), insulin-like growth factor-1 (IGF-1) and transforming growth factor-β (TGF-β) stimulate the chondrocytes to synthesis ECM. In degradative phase, enzymes like matrix metalloproteinases (MMPs) and a disintegrin and metalloproteinase with thrombospondin motif’ (ADAMTS) in the presence of inflammatory cytokines digest the ECM and inhibit its synthesis. Physiologically, there exists a strict regulation of matrix synthesis that leads to a balance between these two phases. However, in OA, the presence of inflammatory cytokines increases the synthesis of proteolytic enzymes, decreases the matrix metalloproteinases inhibitors and reduces the matrix synthesis, causing imbalance in the two phases. In other words, OA is defined by the loss of cartilage tissue through degradation of collagen type II (col II) and proteoglycan components in the ECM [21].

## 4. Autologous Chondrocyte Implantation

Throughout the years, many techniques, including microfracture, microdrilling, abrasion chondroplasty, and debridement, have been developed to promote cartilage regeneration [3]. Disappointingly, treatment with these techniques resulted in the formation of fibrocartilage with poorer mechanical properties compared to original hyaline cartilage. Thus, ACI was introduced to promote hyaline cartilage regeneration. 

ACI is a technique used to repair the damaged cartilage through the implantation of chondrocytes. The procedure involves the collection of cartilage tissue for chondrocyte isolation and expansion in vitro before implanting the cells back to the chondral defect [22]. In the first generation, the implanted chondrocytes are covered with a periosteal flap which is sutured to the surrounding cartilage tissue. The use of periosteal flap has several issues, including the fragile nature of the tissue that renders it difficult to handle during the surgery and periosteal hypertrophy that leads to post-operative failure. Very soon thereafter, the second generation ACI which uses the collagenous scaffold to replace periosteal flap was introduced. The third generation, also known as matrix-associated ACI (MACI), involved the suspension of chondrocytes within a hydrogel scaffold or chondrocytes seeded on a scaffold. In addition, the chondrocytes also can be cultured as a spheroid to stimulate self-secretion of ECM prior transplantation. The application of MACI eliminates the need of a native or synthetic periosteal patch and allows easier control of cell distribution on the defect. More importantly, MACI can be used to treat more severe osteochondral defect [6,23]. 

Key limitations of ACI that have yet to be resolved are the dedifferentiation of expanded chondrocytes and the long culturing period of 2–3 months to fetch the quantity of cells needed. It is well known that chondrocytes expanded in vitro will undergo dedifferentiation which reduces the cells’ capacity to regenerate hyaline cartilage. The characteristic of chondrocytes changed in the presence of different serum supplements. In this review, we will discuss the changes to the chondrocytes when different serum supplements are used. 

## 5. Conventional Expansion of Chondrocytes Using Foetal Bovine Serum

FBS has been used for the expansion of chondrocytes for a long period of time. To prepare FBS, blood is drawn from the foetus of pregnant cows sent to slaughter. When a pregnant cow is discovered in slaughter line, the foetus is separated in the abattoir and the foetal blood is collected under aseptic condition and without anaesthesia. Foetal blood is mostly collected via cardiac puncture to minimize the risk of contamination. Other than that, the blood can also be collected through umbilical vein puncture [24]. 

FBS is rich in important biological molecules such as proteins, lipids, trace elements, glucose, attachment factors, hormones and growth factors that support cell survival and growth [25]. Table 1 summarizes the advantages and disadvantages of FBS, HPL and defined medium in cell culture. Generally, FBS is abundant and relatively cheaper compared to human serum and chemically defined medium. Furthermore, FBS contains most of the biological molecules needed by the cells and is a suitable supplement for most of the human and animal cells. In fact, majority of the studies and publications used FBS. Nonetheless, FBS also has several shortcomings that render it not ideal for cell expansion within the cGMP facility. These shortfalls include poorly defined composition, lot-to-lot variation, and the potential transmission of animal pathogen and carryover of animal proteins. In addition, certain cells, e.g., epithelial cells, do not growth well in the presence of FBS. Finally, the production of FBS is linked with ethical issues related to animal welfare. 

**Figure 1:**
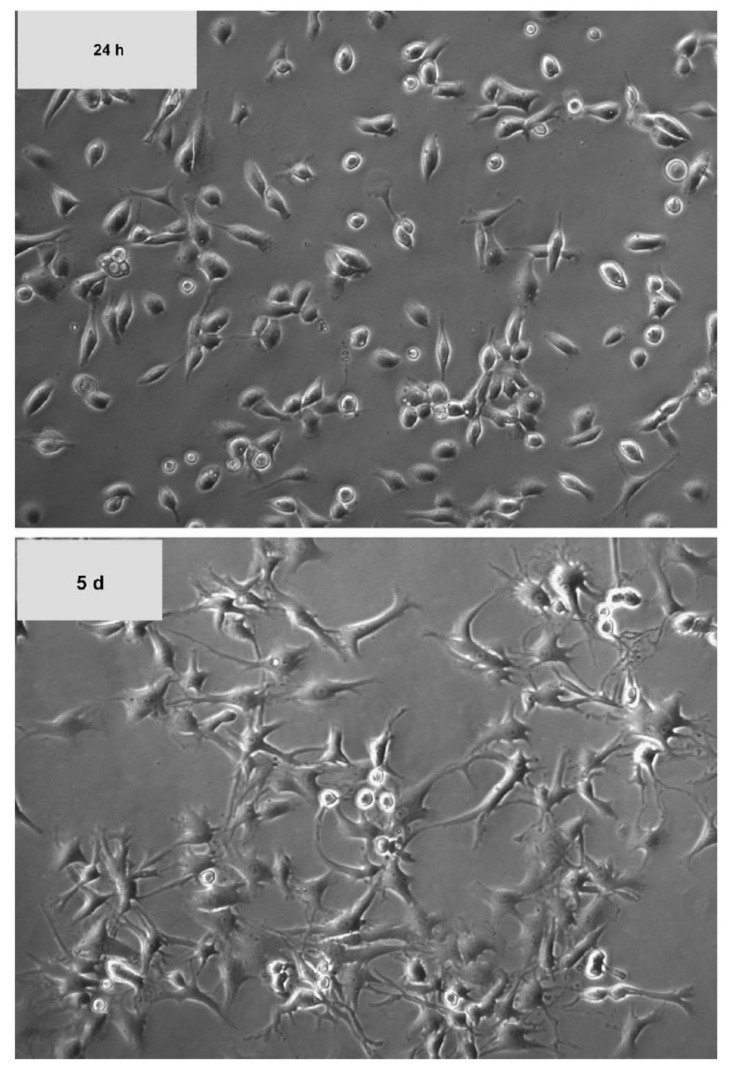
Morphological changes of chondrocytes with time in culture. The chondrocytes become dedifferentiated during in vitro expansion and the morphology gradually changes from rounded and cobblestone to elongated and slender. (reproduced from reference [31]).

Many publications have reported the in vitro expansion of chondrocytes with FBS. Generally, FBS supports the proliferation of chondrocytes but cannot prevent cell dedifferentiation after prolonged culture. In addition, chondrocyte dedifferentiation is accelerated at low cell seeding density and in the presence of biological molecules such as interleukin-1 (IL-1), fibroblast growth factor-2 (FGF-2), and retinoic acid [26,27]. The dedifferentiated chondrocytes lost its rounded, cobblestone structure and acquired a fibroblastic morphology [28]. Figure 1 shows the changes in chondrocyte morphology at day 1 and day 5. In terms of matrix production, dedifferentiated chondrocytes switch from secretion of collagen type II, IX and XI and aggrecan to collagen type I and III [19,28,29]. The loss of these cartilage-specific markers renders the dedifferentiated chondrocytes not ideal for ACI as it will produce unspecific ECM with poor mechanical property. At the same time, dedifferentiated chondrocytes also have lower expression of SOX9, a chondrogenic transcriptional factor [30]. 

## 6. Human Platelet Lysate as Replacement for Foetal Bovine Serum

HPL is used for the expansion of chondrocytes to overcome the setbacks of FBS, especially during the production of chondrocytes in the cGMP facility. Unlike FBS, HPL is derived from human and devoid of the risk of animal pathogen transmission and animal protein contamination. In addition, platelet concentrates used to prepare HPL is readily available and the procedure to prepare HPL is very simple. HPL can be prepared from fresh and expired platelet concentrates and from buffy-coat discarded during the packed red blood cell production [32,33]. Ideally, fresh platelet concentrates are used for the preparation of HPL as platelet storage lesions and degradation may reduce the availability of essential growth factors within the platelet concentrates. Furthermore, it has been suggested that HPL should be prepared using platelets from blood group O donors with plasma from blood group AB donors to reduce the risk of agglutination [33]. It is estimated that 50–60% of the platelet concentrates in blood bank are stored till expired and discarded [34]. These expired platelet concentrates can be collected and used to produce HPL. In a previous study, we have reported the preparation of HPL via repeated freezing and thawing of 3–5 bags of expired platelet concentrates with the same blood group and rhesus that were later pooled and centrifuged to separate the HPL [35]. We were keen to use expired platelet concentrates to avoid competition with blood bank that need the fresh platelet concentrates to save lives. It has been reported that HPL prepared from fresh and expired platelet concentrates were equally potent in enhancing the growth of mesenchymal stem cells despite the sharp drop in platelet count in expired platelet concentrates [36]. 

HPL is rich in growth factors such as platelet-derived growth factor (PDGF), basic fibroblast growth factor (bFGF), transforming growth factor-beta 1 (TGF-β1), insulin-like growth factor-1 (IGF-1), vascular endothelial growth factor (VEGF) and hepatocyte growth factor (HGF) [37,38]. In addition, HPL also contains coagulation factors, adhesion molecules, protease inhibitors, proteoglycans, CCL-5, CXCL 1/2/3, and soluble CD40L [34]. These bioactive factors can modulate the function and proliferation of chondrocytes. 

HPL has been consistently shown to stimulate chondrocyte proliferation [35,39,40,41]. In addition, Muraglia et al. also reported that addition of HPL to FBS supplement significantly enhanced the chondrocyte proliferation [42]. Kachroo et al. compared the expansion of chondroprogenitor cells isolated from cartilage with HPL and FBS [43]. The authors found that chondroprogenitor cells cultured with HPL have higher proliferation but lower expression of chondrogenic (aggrecan and col II), dedifferentiation (col I) and hypertrophic markers (col X) compared to those expanded with FBS. However, just like FBS, HPL cannot prevent the dedifferentiation of cultured chondrocytes. Table 2 shows the list of studies reported HPL-based chondrocyte expansion and their key findings.

## 7. Chondrocyte Dedifferentiation and Redifferentiation of Dedifferentiated Cells

**Figure 2:**
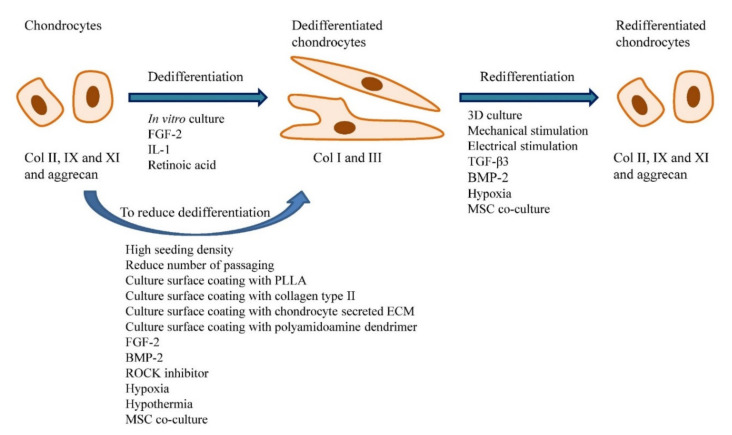
Factors that affect chondrocyte dedifferentiation and redifferentiation in vitro. Chondrocytes cultured in vitro will become dedifferentiated and the process is accelerated in the presence of FGF-2, IL-1 and retinoic acid. Strategies such as seeding the cells at high density, reduce frequency of passaging, culture surface coating with specific substrates (i.e., PLLA, collagen type II, chondrocyte secreted ECM and polyamidoamine dendrimer), addition of specific chemicals (i.e., FGF-2, BMP-2 and ROCK inhibitor), cell culture at low oxygen tension and low temperature, and co-culture with MSCs can reduce chondrocytes dedifferentiation. The dedifferentiated chondrocytes can be redifferentiated through 3D cell culture, application of mechanical and electrical stimulus, addition of certain chemicals (i.e., TGF-β1 and BMP-2), hypoxic culture and MSC co-culture.

The dedifferentiation of chondrocytes in culture can be slowed down by seeding cells at high density, reducing number of cell passage, culturing the cells in hypoxic and hypothermic environment, expanding the cells on specific substrates (e.g., col II, poly(L-lactic acid), polyamidoamine dendrimer), addition of certain chemicals (e.g., insulin-transferrin-selenium, FGF-2, ROCK inhibitor), and co-culturing with mesenchymal stem cells (MSCs) [44,45,46,47,48,49,50,51,52]. Coating of culture surface with collagen type I (col I) and aggrecan showed limited success as they were found to reverse the changes in col I and aggrecan gene expression but failed to reverse the downregulation of col II gene expression [53]. Interestingly, FGF-2 that has been reported to enhance chondrocyte dedifferentiation was also found to slow down the dedifferentiation process [27,47]. Table 3 shows the list of strategies that can be applied to slow down dedifferentiation of chondrocytes in monolayer culture. Figure 2 shows the factors that modulate the chondrocyte dedifferentiation and redifferentiation in vitro.

As chondrocytes dedifferentiate rapidly in culture and the strategies developed only manage to slow down but cannot prevent chondrocyte dedifferentiation, many efforts have been devoted to establishing techniques that redifferentiate the dedifferentiated chondrocytes. The dedifferentiated chondrocytes can be redifferentiated via 3-dimensional (3D) culture. The redifferentiated chondrocytes reacquire the rounded morphology and regain the cartilage-specific markers as well as embedded themselves in cartilage-like matrix [30,60,61]. Interestingly, the redifferentiated chondrocytes retained its rounded morphology and cartilage-like matrix secretion even after the cells were recovered and reseeded in monolayer culture. In addition, dedifferentiated chondrocytes also can be redifferentiated using chondrogenic inducers. An extensive study has been performed to check the combination of 2 factors among the 12 candidate factors, i.e., BMP-2, IGF-1, FGF-2, insulin, IL-1RA, growth hormone, testosterone, 17β-estradiol, parathyroid hormone, L-3,3′,5′-triiodothyronine (T3), α-25-dihydroxy vitamin D3, and dexamethasone, to identify the most potent pair that reverses chondrocyte dedifferentiation [62]. Results showed that combination of BMP-2 and insulin is most potent in promoting chondrocyte redifferentiation. Furthermore, hypoxia, mechanical stimulation, electrical stimulation, chondrocyte-MSC co-culture, chondrocyte-synovium derived stem cell co-culture and chondrocyte-chondrocyte co-culture also have been found to promote chondrocyte redifferentiation [63,64,65,66,67,68,69]. Table 4 shows the list of strategies proven to promote redifferentiation of dedifferentiated chondrocytes.

Several studies have compared the redifferentiation potential of the dedifferentiated chondrocytes cultured with FBS and HPL. Pereira et al. reported that chondrocytes cultured with FBS lost its redifferentiation potential in 3D culture after 3 population doublings and the chondrocytes cultured with HPL maintained its redifferentiation potential even after 10 population doublings [39]. Similarly, Hildner et al. showed that chondrocytes cultured with HPL have better redifferentiation potential compared to those expanded with FBS when comparison was made between cells at the same population doubling number [40]. Contradictory results were reported by Sykes et al. whereby the researchers found that chondrocytes cultured with HPL have poorer redifferentiation potential compared to those cultured with FBS [8]. HPL also has been found to reduce the deposition of cartilaginous matrix by chondrocytes in 3D culture [41]. 

The discrepancies in the results collected are likely due to the different HPL preparation technique used. Thus, an international effort is needed to standardize the HPL preparation technique. Furthermore, standardization on the safety and quality criteria of HPL is also very important. Just like blood donation, the donors/samples must be screened for infectious diseases and the final product needs to be free from any microorganism contamination. Techniques such as UV light and gamma irradiation can be used to inactivate pathogens in HPL. As for the quality, cellular behaviours such as proliferation, differentiation and metabolic activity are parameters to determine the functionality of HPL.

Regarding the potency of the chondrocytes cultured with HPL, thus far, no preclinical and clinical studies have reported the treatment of OA with chondrocytes cultured with HPL. It has been reported that chondrocytes cultured with HPL secrete chondrocyte chemoattractants and stimulate a transient activation and resolution of inflammation which are beneficial for cartilage regeneration [39]. As discrepant results were reported for redifferentiation potential of chondrocyte cultured with HPL and FBS, it remains uncertain on which serum supplement can produce chondrocytes that are more potent for the treatment of OA. The therapeutic potential of chondrocytes cultured with HPL certainly needs to be confirmed in preclinical and clinical studies before it can be used as a routine expansion protocol for chondrocytes. Nonetheless, the usage of HPL can bring down the cost of ACI as it can yield the needed cell number in a shorter period. 

## 8. Other Alternatives for Foetal Bovine Serum

### 8.1. Human Serum

Apart from FBS and HPL, human serum (HS) also has been used to culture chondrocytes. HS has been reported to be superior compared to FBS in promoting chondrocyte proliferation but inferior compared to FBS in preventing chondrocyte dedifferentiation [89]. Chondrocytes cultured with HS showed poorer expression of cartilage-specific col II and proteoglycans in term of number of positive cells and intensity as well as higher expression of col I, a dedifferentiation marker. Furthermore, the study also found that HS is poorer in promoting chondrocyte redifferentiation compared to FBS when the cells were culture in 3D. In a separate study, the authors reported that HS increased the chondrocyte proliferation by eight-fold compared to the FBS [90]. However, the study did not examine the chondrocyte dedifferentiation and redifferentiation. Chua et al. reported that HS is superior compared to FBS in stimulating chondrocyte proliferation and the cell growth can be further enhanced by adding bFGF [91]. No differences were detected in the expression of col I and col II in both serum supplements and good cartilage was formed in the engineered cartilage implanted in nude mice. 

Anderer and Libera reported the culture of chondrocyte spheroid with HS and found that it formed cartilage-like tissue [92]. In contrast, chondrocyte spheroid cultured with FBS demonstrated delayed formation of cartilage-like tissue and presence of a central disintegrated area at three months. Table 5 lists the studies that reported chondrocyte expansion with human serum and their key findings.

### 8.2. Serum-Free Medium

With more understanding on the cellular demand, researchers have developed serum-free medium (SFM) containing defined components essential for the chondrocyte culture to replace the serum supplementation. SFM is generally more expensive and takes a long time to develop as thorough experimentation is needed to identify the cell phenotype and characteristic when the chondrocytes are adapted to a new culture medium. Nonetheless, the cost of SFM is expected to go down in future when the demand increases. The use of SFM has many advantages as it is devoid of batch-to-batch variation, free from xenogeneic and allogenic components, and can support chondrocyte proliferation while reducing cell dedifferentiation. Table 6 presents the studies using SFM to expand chondrocytes. 

Malpeli et al. developed a SFM for the expansion of chondrocytes and found that SFM is more potent in enhancing chondrocyte proliferation and maintaining SOX9 expression compared to FBS [93]. In addition, the chondrocytes expanded with SFM can redifferentiate spontaneously when cultured in 3D whilst those cultured with FBS required induction factors to redifferentiate. Similarly, Ho et al. reported that SFM increased chondrocyte proliferation and the cell expanded with SFM showed more potent redifferentiation in 3D culture compared to those expanded with FBS [96]. Furthermore, the chondrocytes cultured with SFM also showed lower expression of col X, a chondrocyte hypertrophy marker, compared to those expanded with FBS upon redifferentiation. Shao et al. found that SFM is more potent in maintaining the chondrocyte differentiation markers compared to FBS and is on par with FBS in promoting cell proliferation [95]. At the same time, the authors also found no difference in chondrocyte redifferentiation potential in 3D culture with both mediums. Steward et al. reported that SFM is more potent compared to FBS in maintaining the chondrocyte expression of col II and aggrecan as well as more effective in suppressing the expression of col I [99]. In a recent study, SFM has been found to be superior in maintaining the SOX9 expression of chondrocytes cultured in 3D [94].

Contradictory results have been reported by Martinez et al. which showed that chondrocytes cultured in hypoxic 3D environment and nourished with SFM have poorer redifferentiation compared to those nourished with HS [97]. In a separate study, the authors found that SFM is on par with FBS in promoting chondrocyte proliferation [98]. However, SFM lowered the expression of col I compared to FBS and a higher reduction is recorded in SFM with lower calcium concentration.

### 8.3. Others

Jeyakumar et al. used platelet-rich plasma (PRP) and hyperacute serum (HAS) to expand human chondrocytes [81]. PRP is a fraction of blood rich in platelet and HAS is the serum separated from the platelet-rich fibrin (PRF) clot during the preparation of PRF [100,101,102]. HAS supplementation gave the highest cell proliferation at day 9 compared to PRP and FBS. However, PRP is most potent in preventing chondrocyte dedifferentiation compared to HAS and FBS in both normoxic and hypoxic conditions. Similar results have been reported by Akeda et al. which showed that PRP significantly increased the proliferation and enhanced the expression of col II and proteoglycan of porcine chondrocytes cultured in alginate beads compared to platelet-poor plasma (PPP) and FBS [103]. Drengk et al. reported contradict results whereby they found that PRP only promoted the proliferation of 6/10 ovine chondrocyte samples and PRP was not as potent as FBS in promoting the expression of col II in chondrocytes cultured as micromass [104].

In a study comparing the chondrocyte expansion with bone marrow extract and FBS, the authors found that bone marrow extract is inferior in promoting human chondrocyte proliferation, but superior in preventing cell dedifferentiation compared to FBS [105].

## 9. Conclusions

HPL is superior compared to FBS in promoting chondrocyte proliferation in vitro. Discrepancy in the capability of HPL in maintaining chondrocyte chondrogenic potential, i.e., reduces chondrocyte dedifferentiation and enhances chondrocyte redifferentiation capacity, are likely due to the differences in HPL preparation methods. From a practical perspective, further research to develop a standardized HPL preparation method that can produce HPL which can maintain the chondrocyte chondrogenic potential reproducibly is therefore warranted. As for the defined medium, the high cost and lack of GMP-grade defined medium hinder its usage in culturing chondrocytes for research and clinical purposes.

## Figures and Tables

**Table 1:** Pros and cons of FBS, HPL and defined medium in cell culture.

	FBS	HPL	Defined Medium
**Pros**	Abundant and easily available	Easy to produce	Components are well-defined
	Cheaper compared to HPL and chemically defined medium	A universal growth supplement that is suitable for most human and animal cells	No batch-to-batch variation
	A universal growth supplement that is suitable for most human and animal cells	Contains most of the factors required for cell survival and proliferation	No risk of disease transmission
	Contains most of the factors required for cell survival and proliferation	No risk of xenogeneic immune reaction	No risk of xenogeneic/allogeneic protein contamination
	Used by most of the studies and publications	Can be prepared using autologous blood	No unintended interaction with test substances
**Cons**	Components are ill-defined	Components are ill-defined	Not available for certain cells
	Batch-to-batch variation	Batch-to-batch variation (reduced by pooling)	Some defined medium required extra coating to promote cell attachment
	A potential source of animal microbial contaminants	Unintended interaction with test substances	More expansive compared to FBS and HPL
	Risk of animal protein contamination on cells prepared for clinical usage	Fewer vendors distribute the product	Time consuming and difficult to develop
	Unintended interaction with test substances	Potential eliciting allogeneic immune response	
	Ethical concerns with animal welfare	Risk of transmitting human viruses	

**Table 2:** Key findings in the studies reported HPL-based chondrocyte expansion.

References	Source of Chondrocytes	Proliferation	Dedifferentiation	Redifferentiation	Other Key Findings
[35]	Human	HPL increases chondrocyte proliferation compared to FBS.	HPL and FBS fail to prevent chondrocyte dedifferentiation.	-	Chondrocytes cultured with HPL have lower expression of col X, a chondrocyte hypertrophic marker compared to those cultured with FBS.
[39]	Human	HPL increases chondrocyte proliferation compared to FBS.	HPL and FBS fail to prevent chondrocyte dedifferentiation.	Chondrocytes cultured with HPL maintains its redifferentiation potential after 10 population doublings and those cultured with FBS lost its redifferentiation potential after 3 population doublings.	Chondrocytes cultured with HPL in the presence of IL-1α demonstrate a transient activation and resolution of inflammation. Chondrocytes cultured with HPL secrete chondrocyte chemoattractants.
[8]	Human	HPL increases chondrocyte proliferation compared to FBS.	-	Chondrocytes cultured with HPL have poorer redifferentiation potential.	-
[40]	Human	HPL increases chondrocyte proliferation compared to FBS.	-	At the same population doubling number, chondrocytes cultured with HPL show better redifferentiation potential compared to those cultured with FBS.	-
[41]	Bovine	HPL increases chondrocyte proliferation compared to FBS.	-	-	Chondrocytes cultured in 3D with HPL showed poorer formation of cartilaginous matrix compared to those cultured with FBS.
[42]	Human	Addition of HPL to FBS promotes chondrocyte proliferation compared to FBS alone.	-	-	Chondrocytes cultured in 3D with FBS + HPL form cartilage-like tissue.
[43]	Human	HPL increases chondroprogenitor cell proliferation compared to FBS.	-	-	Chondroprogenitor cells cultured HPL have lower expression of col I, II, X, and aggrecan compared to those expanded with FBS.

HPL—Human platelet lysate, FBS—Foetal bovine serum, 3D—3-dimensional, col X—Collagen type X.

**Table 3:** Strategies to reduce dedifferentiation of cultured chondrocytes.

Strategy	Specific Factor	References
Seeding density	High seeding density	[54]
Cell passaging	Reduce number of passaging	[50]
Surface substrate	Polyamidoamine dendrimer	[46]
	PLLA	[44]
	Chondrocytes secreted ECM	[55]
	Collagen type II	[56]
Chemical compound	Insulin-transferrin-selenium	[45]
	FGF-2	[47]
	BMP-2	[57]
	ROCK inhibitor	[48]
Hypoxia	2% O_2_	[49]
	1.5% O_2_	[58]
Hypothermia	32.2 °C	[52]
Co-culture	Ratio of chondrocytes: MSCs; 2:1	[51]
	Ratio of chondrocytes: MSCs; 1:1	[59]

**Table 4:** Strategies to promote redifferentiation of dedifferentiated chondrocytes.

Strategy	Specific Factor	References
3D culture	Barium-alginate nanofiber	[30]
	Alginate bead	[60]
	Cell pellet	[61]
	Polyethylene glycol hydrogel	[70]
	Agarose hydrogel	[71]
	Hyaluronan hydrogel	[72]
	Hyaluronic acid methyacrylate hydrogel	[73]
Mechanical stimulation	Cyclic sinusoidal dynamic tensile mechanical stimulation	[65]
	Shear and/or compression stress and gelatin methacryloyl/hyaluronic acid methacrylate	[74]
	Intermittent hydrostatic pressure and alginate bead	[75]
	Spinner flask	[76]
Electrical stimulation	Capacitively coupled electric field stimulation	[66]
	Alternating electrical stimulation and hypoxia	[77]
Chemical compound	TGF-β3 and high seeding density	[78]
	BMP-2 and alginate bead	[79]
	BMP-2 and insulin	[62]
	TGF-β1 and dexamethasone	[80]
	Platelet-rich plasma	[81]
Hypoxia and 3D culture	5% O_2_ and alginate bead	[82]
	1% and 5% O_2_ and MPEG-PLGA scaffold	[83]
3D co-culture	Ratio of chondrocytes: MSCs; 3:7	[67]
	Ratio of chondrocytes: MSCs; 1:3	[84]
	Ratio of chondrocytes: MSCs; 1:3	[85]
	Ratio of chondrocytes: MSCs; 1:1	[86]
	Chondrocyte pellet on MSCs-laden chitosan/β-glycerophosphate hydrogel	[87]
	Ratio of chondrocytes: synovium derived stem cells; 1:1	[68]
	Ratio of chondrocytes: primary chondrocytes; 4:1	[88]
	Ratio of chondrocytes: primary chondrocytes; 4:1	[69]

**Table 5:** Key findings in the studies reported human serum-based chondrocyte expansion.

References	Source of Chondrocytes	Proliferation	Dedifferentiation	Redifferentiation	Other Key Findings
[89]	Human	HS increases chondrocyte proliferation compared to FBS.	HS is inferior compared to FBS in preventing chondrocyte dedifferentiation.	HS is inferior compared to FBS in promoting chondrocyte redifferentiation.	-
[90]	Human	HS increases chondrocyte proliferation compared to FBS.	-	-	-
[91]	Human	HS increases chondrocyte proliferation compared to FBS.	HS and FBS are comparable in maintenance of chondrocyte markers.	-	Supplement of 5 ng/mL bFGF enhances chondrocyte proliferation. The HS expanded chondrocytes form good cartilage in vivo.
[92]	Human	HS increases chondrocyte proliferation compared to FBS.	-	-	Substitution of HS with FBS delayed cartilage-like tissue formation and a hollow central area is formed at 3 months.

HS—Human serum, FBS—Foetal bovine serum, bFGF—Basic fibroblast growth factor.

**Table 6:** Key findings in the studies reported chondrocyte expansion with serum-free medium.

References	Medium	Source of Chondrocytes	Proliferation	Dedifferentiation	Redifferentiation	Other Key Findings
[93]	Coon’s modified Ham’s F12 medium with 5 ng/mL each of FGF-2, PDGF-bb, and EGF, 5 µg/mL insulin, 10^−8^ M dexamethasone, 50 µg/mL ascorbic acid, 50 µg/mL human transferrin, 2% human serum albumin, 6.25 µM linoleic acid, 30 µg/mL cholesterol, 5 × 10^−5^ M 2-mercaptoethanol, 30 nM selenium, 33 µM biotin, and 17 µM sodium pantothenate	Human	SFM increases chondrocyte proliferation compared to FBS.	SFM preserves the SOX9 expression better than FBS.	Chondrocytes cultured with SFM can redifferentiate spontaneously in 3D culture while those expanded with FBS requires induction factors to redifferentiate.	-
[94]	DMEM medium with 1% ITS-A, 0.4 µM proline, 50 µg/mL ascorbic acid, 10 mM HEPES, 0.1 mM non-essential amino acids, 2 mM L-glutamine and 1% antibiotic-antimycotic	Human	-	SFM preserves the SOX9 expression of 3D cultured chondrocytes better than FBS.	-	-
[95]	DMEM/F12 medium with 10 ng/mL FGF-2 and 10 ng/mL PDGF. DMEM/F12 medium with 10 ng/mL FGF-2 and 10 ng/mL IGF-1.	Bovine	Chondrocytes expanded with SFM and FBS have similar pace of cell proliferation.	SFM is superior compared to FBS in preventing chondrocyte dedifferentiation.	Chondrocytes expanded with SFM and FBS have similar redifferentiation potential in 3D culture.	-
[96]	DMEM/F12 medium with 10% Knockout SR, 2 ng/mL FGF-2, 2 ng/mL PDGF-AB, 2 ng/mL EGF and 10^−8^ M dexamethasone	Human	SFM increases chondrocyte proliferation compared to FBS.	-	Chondrocytes cultured with SFM show better redifferentiation in 3D culture compared to those expanded with FBS.	Chondrocytes cultured with SFM have lower expression of col X compared to those expanded with FBS upon redifferentiation.
[97]	Tissue SS supplements for biotechnology (Medi-Cult A/S)	Human	-	-	Chondrocytes expanded with SFM shows poorer redifferentiation in 3D culture compared those cultured with HS in hypoxic environment.	-
[98]	DMEM/F12 medium with 0.4 mM proline, 1.5 mM glutamine, 22 mM sodium bicarbonate, 8.9 mg/L alanine, 15 mg/L asparagine, 13.3 mg/L aspartic acid,14.5 mg/L glutamic acid, 7.5 mg/L glycine, 11.5 mg/L proline, 10.5 mg/L serine, 12.5 mM HEPES, 1× penicillin/streptomycin, 10 µg/mL insulin, 5.5 µg/mL transferrin, 0.05% (*w*/*v*) bovine serum albumin, 1.7 mM linoleic acid, 0.5 µg/mL sodium selenite, 5 × 10^−5^ M 2-mercaptoethanol and 10^−8^ M dexamethasone	Bovine	Chondrocytes expanded with SFM and FBS have similar pace of cell proliferation.	SFM lowers the expression of col I compared to FBS.	-	Lower calcium concentration in SFM is favourable to reduce expression of col I.
[99]	Opti-MEM (Gibco)	Equine	-	Chondrocytes cultured with SFM have higher expression of col II and aggrecan as well as lower col I compared to those cultured with FBS.	-	-

SFM—Serum-free medium, FBS—Foetal bovine serum, HS—Human serum, 3D—3-dimensional, FGF2—Fibroblast growth factor 2, PDGF bb—Platelet-derived growth factor bb, EGF—Epidermal growth factor, col I—Collagen type I, col II—Collagen type II, col X—Collagen type X.

## Data Availability

Not applicable.
